# Endoscopic Ultrasound-Lavage Technique for Pancreatic Cancer: An In Vivo Pilot Study

**DOI:** 10.3390/diagnostics16020230

**Published:** 2026-01-11

**Authors:** Takahiro Abe, Masayuki Kato, Nana Shimamoto, Tomotaro Komori, Naoki Matsumoto, Takafumi Akasu, Masafumi Chiba, Masanori Nakano, Kimio Isshi, Yuichi Torisu, Kazuki Sumiyama

**Affiliations:** 1 Department of Endoscopy, The Jikei University School of Medicine, Tokyo 105-8471, Japan; masakato89@gmail.com (M.K.); nanahachinetyou@yahoo.co.jp (N.S.); ccl09720@gmail.com (M.C.); isshi-ki@outlook.jp (K.I.); kaz_sum@jikei.ac.jp (K.S.); 2 Division of Gastroenterology and Hepatology, Department of Internal Medicine, The Jikei University School of Medicine, Tokyo 105-8471, Japan; tarokomo@gmail.com (T.K.); matsumotonaoki621@gmail.com (N.M.); tkfm0902aks@gmail.com (T.A.); masanori-nakano@jikei.ac.jp (M.N.); tori3991@gmail.com (Y.T.)

**Keywords:** pancreatic cancer (PC), endoscopic ultrasound (EUS), EUS-guided fine-needle aspiration (EUS-FNA), EUS-lavage technique, EUS-LT, peritoneal lavage, EUS-lavage technique, staging laparoscopy (SL), porcine model

## Abstract

**Background**: Pancreatic cancer (PC) has a very poor 5-year survival and prognosis. Even when CT or MRI shows no metastasis, staging laparoscopy(SL) still detects tiny peritoneal deposits in 20–30% of patients, making them ineligible for surgery. SL is invasive, requiring general anesthesia and substantial resources. Endoscopic ultrasound (EUS) allows the observation of the bile ducts, pancreas, and abdominal cavity, and EUS-guided fine-needle aspiration (EUS-FNA) is essential for pathological diagnosis. Reports on using EUS to perform peritoneal lavage cytology are currently not available. We hypothesized that combining EUS-FNA with peritoneal lavage (EUS-lavage technique; EUS-LT) could enhance staging accuracy and avoid unnecessary surgical procedures. **Methods**: Ten in vivo porcine models underwent EUS-LT. Using a 19G FNA needle, 800 mL saline was instilled into the intraperitoneal cavity and then recovered. Two refinements were introduced sequentially: an ENBD catheter with additional side holes and, subsequently, a side-hole introducer (EndoSheather) that eliminated balloon dilation. The primary endpoint was procedural success. Secondary endpoints included safety, complications, recovered volume, duration of endoscopic procedure, and time required to instill 800 mL. Nonparametric tests compared outcomes across iterations. **Results**: Ten-model porcine in vivo model series were included, and all procedures were successful. No device malfunctions or unanticipated technical failures; one minor mucosal injury during saline injection resolved after re-puncture. The average procedure time was 31.1 min. Stepwise refinements shortened procedure and infusion times and increased recovered volume. Recovered volume approached the instilled amount in later cases, indicating efficient performance. **Conclusions**: In this ten-model in vivo series, EUS-LT demonstrated technical feasibility and short-term safety.

## 1. Introduction

Pancreatic cancer (PC), which has a very low 5-year survival rate (2–9%) worldwide, continues to have a very unfavorable prognosis [1,2,3]. Pancreatic imaging modalities, including CT, MRI, and endoscopic ultrasound (EUS), play a central role in lesion detection, disease staging, and treatment planning for pancreatic cancer [4,5,6,7,8]. However, surgery is not a suitable option for most patients diagnosed with PC due to local invasion or distant metastasis at the time of diagnosis. Even when CT or MRI indicates absence of metastasis, preoperative staging laparoscopy (SL) reveals tiny peritoneal deposits in 20–30% of cases, rendering these patients unresectable [9,10,11,12,13]. SL not only has the ability to detect tiny peritoneal metastases but also enables examination of the abdominal cavity before surgery, washing it with saline, and collecting samples to confirm peritoneal metastasis [14]. SL is an invasive procedure requiring general anesthesia and substantial medical resources. A previously published report has demonstrated that percutaneous peritoneal cytology, performed prior to surgery for gastric cancer or PC, has shown safety, sensitivity, and specificity and was comparable to laparoscopic lavage [15]. However, percutaneous peritoneal cytology is also performed under general anesthesia, requiring hospital admission and additional time beyond that needed for pathological evaluation. EUS is an excellent imaging technique that enables observation of the bile ducts, pancreas, and the abdominal cavity through the stomach or the duodenum [8]. EUS-guided fine-needle aspiration (EUS-FNA) is essential for the pathological diagnosis of PC [16,17,18,19,20]. Current ESGE and AGA guidelines recommend EUS-FNA as a first-line diagnostic tool for pancreatic masses [21]. Around the same period when EUS was first introduced, EUS-guided drainage for pancreatic pseudocysts was also reported [22]. Since then, the therapeutic applications of EUS have greatly expanded, and various interventional techniques such as EUS-guided biliary drainage (EUS-BD) and EUS-guided gallbladder drainage (EUS-GBD) have been developed in clinical practice [23,24].

We developed a novel endoscopic ultrasound-guided lavage technique (EUS-LT) that integrates standard EUS-guided fine-needle access with controlled intraperitoneal saline instillation and fluid retrieval. Unlike conventional EUS-guided paracentesis, which is limited to aspiration of pre-existing ascites, EUS-LT is designed to actively create and recover lavage fluid, conceptually analogous to peritoneal lavage performed during staging laparoscopy. EUS-LT is intended as a diagnostic staging approach rather than a therapeutic intervention. By combining established EUS-FNA techniques with intraperitoneal lavage, this approach has the potential to expand the diagnostic capabilities of EUS-based staging while reducing the need for surgical procedures. Specifically, this approach is intended to detect occult peritoneal carcinomatosis in patients with pancreatic cancer who have no radiological evidence of distant metastasis, rather than to treat peritoneal disease.

In the present study, we focused on evaluating the technical feasibility and safety of EUS-LT using an in vivo porcine peritoneal lavage model as a preclinical proof of concept.

## 2. Materials and Methods

### 2.1. Study Design

This preclinical trial was conducted using ten domestic porcine models weighing between 35 and 45 kg. These animals were selected because their abdominal anatomy and peritoneal space closely resemble those of humans, making them suitable for evaluating the technical feasibility and safety of the EUS-guided lavage technique. At the beginning of each experiment, a total of 800 mL of sterile saline was injected into the intraperitoneal cavity to simulate the presence of ascites and to provide sufficient fluid for subsequent lavage assessment. Aspiration of the irrigated fluid was performed using an endoscopic ultrasound-guided lavage technique (EUS-LT). A 19G fine-needle aspiration (FNA) needle was advanced into the peritoneal cavity under real-time EUS guidance to ensure precise access. Once correct needle placement was confirmed, the lavage fluid was aspirated using an endoscopic nasobiliary drainage (ENBD) tube or, in selected cases, the EndoSheather device. All experimental procedures were performed under standardized anesthetic management to minimize discomfort and ensure consistency across animals. The study protocol, including all procedures related to animal handling, anesthesia, fluid infusion, and lavage, was reviewed and approved by the Institutional Animal Care and Use Committee (IACUC) of Jikei University School of Medicine. The entire study was conducted in strict accordance with the ARRIVE guidelines for preclinical animal research, ensuring ethical conduct and methodological rigor (protocol number 2024-082).

### 2.2. Animal Preparation

All animals fasted 24 h before the procedure, however, they had unrestricted access to water. Preanesthetic medication consisted of intramuscular midazolam (0.2 mg/kg; Dormicum; Astellas Pharma Inc., Tokyo, Japan) and medetomidine hydrochloride (60 μg/kg; Domitor; Nippon Zenyaku Kogyo Co., Ltd., Fukushima, Japan), followed by intravenous administration of midazolam at a dose of 0.3 mg/kg. By intubating the animals with an endotracheal tube, they were placed on mechanical ventilation. General anesthesia was sustained through the inhalation of 1–3% isoflurane (Forane; AbbVie GK, Tokyo, Japan). One week after completion of the procedure, following sedation with midazolam, the animals were euthanized through an overdose administration of potassium chloride (1–2 mmol/kg; Terumo Tokyo Japan). Necropsy was performed, and the puncture site of the needle as well as the status of the lavage were examined. In addition, during necropsy, the same puncture procedure was performed, and a tube was inserted and placed to confirm that the tip of the tube was correctly positioned within the abdominal cavity.

### 2.3. Procedure

**Figure 1:**
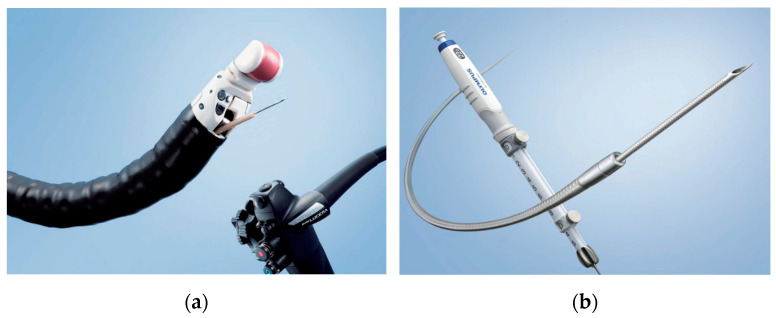
(**a**) Olympus UCT260. (**b**) 19G FNA needle (EZShot3Plus: Courtesy of Olympus Marketing, Inc. Tokyo, Japan).

**Figure 2:**
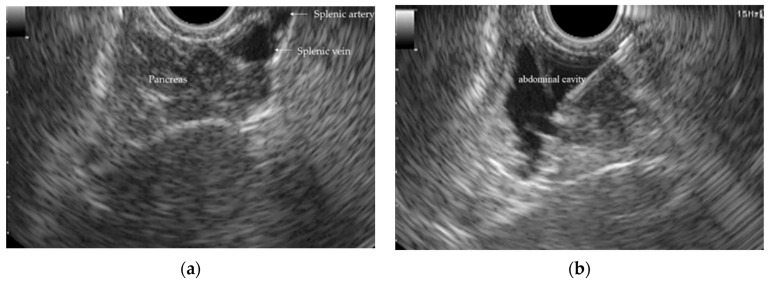
EUS image by detecting the abdominal cavity (**a**) Detecting the omental bursa located between the stomach and pancreas from the EUS image (**b**) Normal saline solution was injected into the peritoneal cavity.

**Figure 3:**
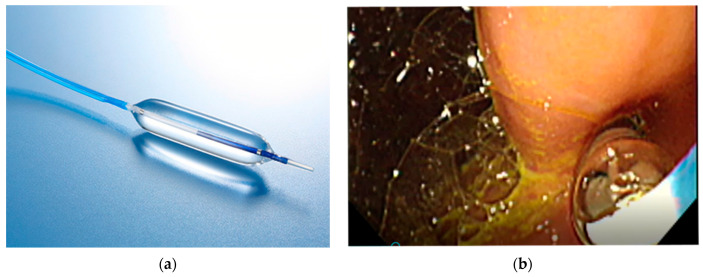
Balloon dilation. (**a**) The 6 mm balloon catheter (REN biliary balloon catheter, KANEKA, Osaka, Japan). (**b**) Inserting through the guide wire.

**Figure 4:**
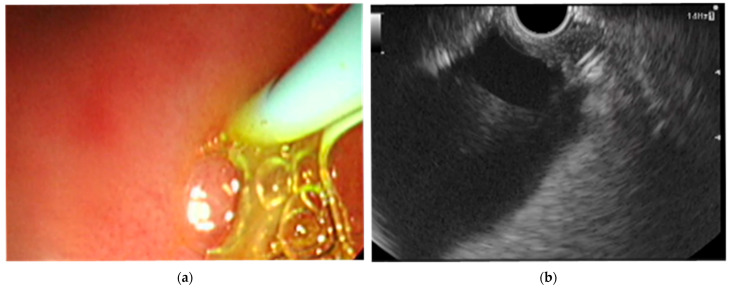
ENBD tube was inserted into the abdominal cavity. (**a**) Endoscopic view. (**b**) Endoscopic ultrasound (EUS) view.

**Figure 5:**
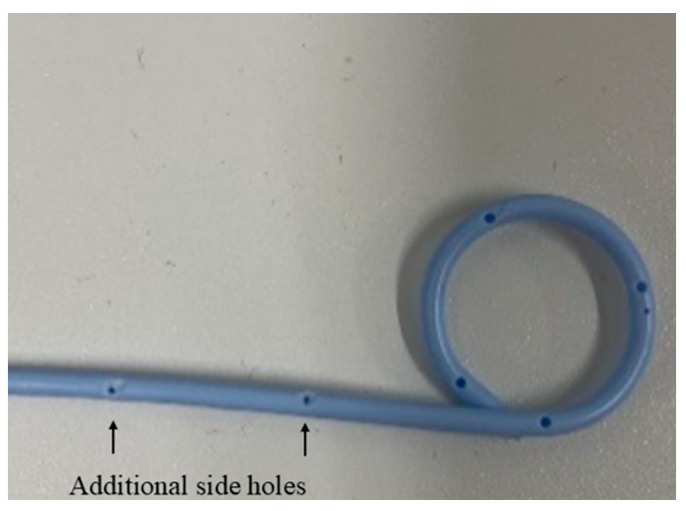
Creating additional holes in the ENBD tube.

**Figure 6:**
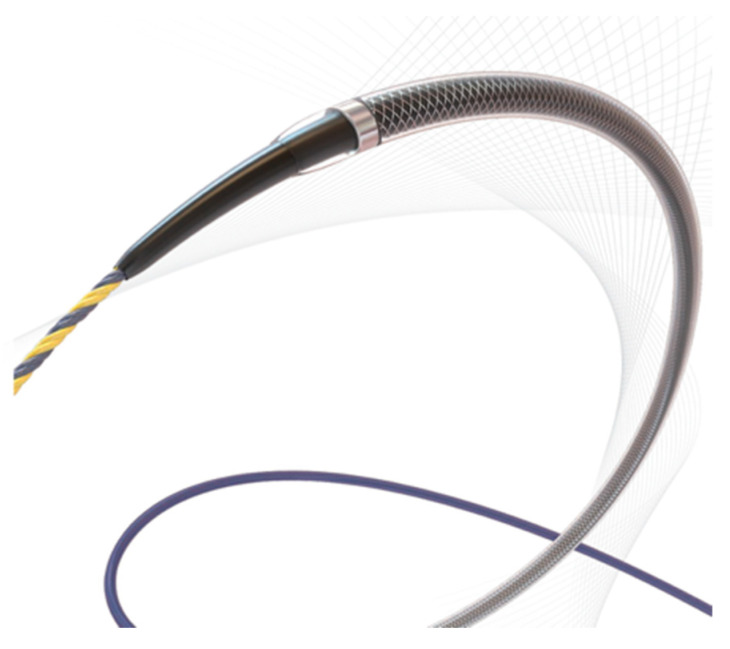
In cases 7–10, the EndoSheather was utilized.

**Figure 7:**
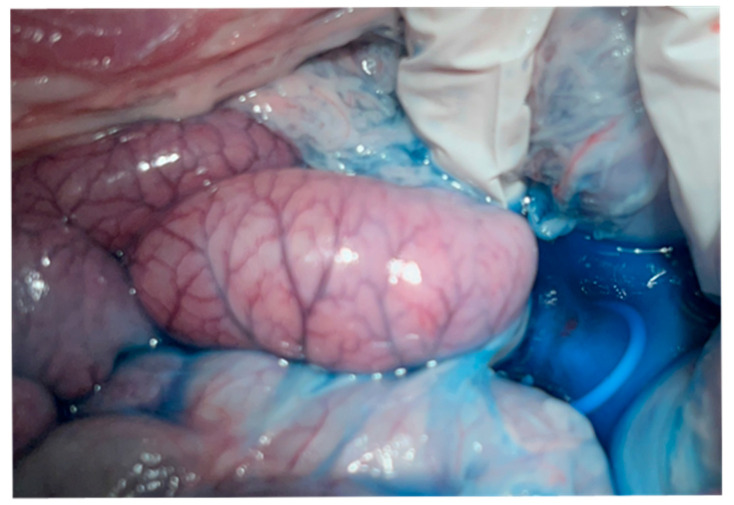
Reverification of the lavage through the ENBD tube from the original puncture site.

1. Passage through the pig’s pharynx (piriform recess) was anatomically challenging for the EUS scope (UCT-260). Therefore, a standard upper endoscope was used to advance and place an overtube (TOP, Tokyo, Japan) into the esophagus, and the EUS scope was then inserted through the positioned overtube (Figure 1).
2. After inserting the endoscope into the stomach, the pancreas was first visualized in the ultrasound mode. Although the porcine pancreas is thinner and more technically difficult to visualize compared with that of humans, placing the probe against the posterior gastric wall allowed for the identification of the aorta and the celiac artery, as well as the splenic artery and splenic vein. Because, as in humans, the porcine pancreas lies along the course of the splenic artery and vein, these vessels served as useful landmarks for its visualization. The omental bursa is located between the stomach and the pancreas (Figure 2).
3. The abdominal cavity of the porcine model was punctured from the stomach wall with a 19G FNA needle (EZShot3 Plus, Olympus Medical Systems, Tokyo, Japan) attached to a syringe filled with normal saline solution to insert An Olympus EUS endoscope (GF-UCT260 with ultrasound processor EU-ME 2, Olympus Medical Systems, Tokyo, Japan) (Figure 1). Fluoroscopic guidance was not used during the procedures.
4. Immediately after detecting the omental bursa located between the stomach and pancreas from the EUS image, under negative pressure, the normal saline solution in the syringe was drawn into the abdominal cavity (Figure 2a).
5. Approximately 100 mL of normal saline solution was injected into the peritoneal cavity to insert the guide wire in the abdominal cavity (Figure 2b).
6. Case 1–6: The FNA needle was removed and the puncture site was dilated using a 6 mm balloon catheter (REN biliary balloon catheter, KANEKA, Osaka, Japan) inserted through the guide wire (Figure 3a,b). Cases 7–10: Balloon dilation was not performed.
7. In cases 1–6, a 6-Fr ENBD tube was advanced into the abdominal cavity (Figure 4a,b), followed by the instillation of approximately 800 mL of warm normal saline. The irrigation saline was recovered as much as possible and the volume recovered was measured. In cases 3-6, additional holes were created in the ENBD tube (Figure 5). In cases 7–10, an EndoSheather (PIOLAX, Yokohama, Japan) was inserted instead of the ENBD tube, and normal saline was injected and subsequently aspirated through it (Figure 6).
8. After removal of the ENBD tube or EndoSheather, the puncture site in the stomach was closed using an endoscopic reopenable clip (SureClip; Micro-Tech, Nanjing, China).
9. Water intake was initiated on the day of the procedure, and feeding was resumed the following day. All experimental pigs were monitored for any decrease in appetite and other signs of clinical deterioration.
10. Necropsy was performed one week following the procedure. The ENBD tube was inserted under the guidance of the clip in the stomach and its position within the abdominal cavity was checked to ensure that it was correctly placed. The accumulation of lavage water was reconfirmed using indigo carmine (Figure 7).

### 2.4. Study Endpoints

The primary endpoint was the success of the procedure. The secondary endpoints included were safety, efficacy, complications, recovered volume of lavage water, duration of endoscopic procedure, and time required for the injection of 800 cc of warm saline solution. The duration of the endoscopic procedure was defined as the time from puncture with the FNA needle until closure of the punctured side hole in the stomach using clips.

## 3. Results

In total, ten porcine peritoneal lavage models were included in the present study. These animals were selected to evaluate the feasibility, safety, and technical performance of our newly developed lavage technique in a controlled experimental setting. Detailed baseline characteristics—including body weight (kg), procedural success, intraoperative and postoperative complications, procedure time (minutes), infusion time (minutes), injection volume (mL), and the amount of recovered lavage fluid (mL)—are summarized in Table 1. All procedures were completed successfully, demonstrating the reproducibility and relative simplicity of the technique even in its early stages of development. Only one case experienced a procedural deviation: during the injection phase, the saline solution was inadvertently delivered into the submucosal layer, resulting in a mild mucosal injury. Despite this event, the injury was limited in extent, did not progress during the procedure, and allowed for successful completion through a repuncture. This incident highlights the importance of appropriate needle positioning but also underscores the flexibility of the method and its capacity to be safely continued after minor adjustments. No additional intraoperative complications were observed, suggesting overall procedural safety. As the study progressed, iterative modifications were introduced to optimize the lavage technique. In cases 3, 4, 5, and 6 (Group 2), we implemented a more efficient strategy by modifying the ENBD tube used for aspiration. Specifically, three side holes were added to the tip of the tube at predetermined positions. This refinement was designed to enhance both the dispersion of the injected saline and the efficiency of its retrieval. The modification proved effective, as evidenced by a notable decrease in injection time and a substantial increase in the recovered lavage volume. These findings suggest that strategic alterations to commonly used devices can significantly improve suction dynamics and procedural efficiency. Further technical advancements were incorporated in cases 7, 8, 9, and 10 (Group 3). After the guidewire was placed, an EndoSheather (PIOLAX, Yokohama, Japan) equipped with a lateral aperture was introduced into the abdominal cavity. This device allowed for more controlled infusion and recovery of saline compared to the modified ENBD tube and provided improved stability during catheter manipulation. Approximately 800 mL of saline was infused into the abdominal cavity of each animal, a volume selected to sufficiently distend the peritoneal space without imposing undue physiological stress. Across these four animals, an average of 66 mL of lavage fluid was successfully recovered. Although this accounted for only a fraction of the total infused volume, it nevertheless represented a substantial improvement in retrieval efficiency compared with earlier procedural attempts. To systematically evaluate differences among the evolving techniques, continuous variables were compared across the three groups—Group 1 (cases 1–2), Group 2 (cases 3–6), and Group 3 (cases 7–10)—using the Kruskal–Wallis test. Given the small sample size and the non-normal distribution of the data, nonparametric methods were considered most appropriate. When significant overall differences were identified, pairwise comparisons were performed using the Mann–Whitney U test with Holm’s correction to control for multiple testing (Table 2). Descriptive statistics are presented as medians with interquartile ranges to accurately reflect data variability. The statistical analysis revealed several important findings. First, both procedure time and infusion time were significantly longer in Group 1 compared with Groups 2 and 3 (all *p* < 0.05). This observation likely reflects an initial procedural learning curve, as well as the efficiency gains achieved through device modification in later groups. Interestingly, no significant differences were observed between Groups 2 and 3 for these parameters, suggesting that while the two later techniques differ in their mechanics, they yield comparable improvements in overall workflow. Second, the recovered lavage volume was significantly lower in Group 1 compared with both Group 2 and Group 3 (*p* < 0.05), indicating that the early procedural strategy was less effective for fluid retrieval. The optimized ENBD tube and the EndoSheather device both substantially improved recovery, although no significant difference was noted between these two groups. This finding may indicate that both modifications enhance aspiration dynamics to a similar degree, despite the different mechanisms by which they operate. Although body weight tended to be higher in Group 1, this factor did not reach statistical significance and is therefore unlikely to have influenced procedural outcomes. However, it is important to note that even modest differences in animal size can affect peritoneal volume and pressure dynamics, and future studies with larger sample sizes may help clarify whether body habitus plays any role in lavage efficiency. No significant intraoperative or postoperative complications occurred in any of the animals after completion of the procedures. The mean procedure time for all cases was 31.1 min, highlighting the practicality and relatively short duration of the technique. Furthermore, the absence of postoperative complications suggests that the lavage procedure is minimally invasive and unlikely to induce delayed adverse effects in this animal model. These findings collectively support the feasibility and safety of the lavage system and suggest its potential applicability in future clinical or translational research settings. Overall, the progressive improvements observed across the three procedural groups provide insight into both the adaptability of the lavage technique and its capacity for refinement. The iterative modifications introduced throughout the study contributed meaningfully to enhanced efficiency and recovery performance without compromising safety.

## 4. Discussion

In this animal study, we evaluated the feasibility and the safety of the EUS-lavage technique (EUS-LT). The effectiveness of the EUS-guided abdominal paracentesis (EUS-P) for cancer staging has previously been reported. The presence of malignant ascites indicates a more advanced stage of cancer [25,26,27]. EUS-LT was modified with respect to EUS-P and percutaneous puncture [7]. The lavage volume of 800 mL was selected based on prior percutaneous peritoneal lavage studies in which the same volume was used and reported to be effective [15]. A previously conducted study had performed EUS puncture for ascitic fluid using a 22 G FNA needle to puncture the abdominal cavity, and had found the procedure to be effective in 11 cases. The average amount retrieved was 14.1 mL, with a range of 0.5–38 mL [28]. However, in the current study, a 19 G needle was used to collect more physiological saline solution. Since a small FNA needle with a hole at its tip could not efficiently retrieve the lavage fluid, an ENBD tube with several holes was used. The normal ENBD tube could aspirate only a small volume of fluid (8–10 mL). Therefore, by creating three additional side holes in the ENBD tube for cases 3, 4, 5 and 6, the injection time was significantly shortened and the volume of lavage fluid collected significantly increased (ranging from 13.5 to 170 mL). In cases 7, 8, 9, and 10, following guidewire placement, the EndoSheather with an additional side hole was advanced into the abdominal cavity to collect physiological saline. This modification permitted the procedure to be performed more smoothly, increased the volume of fluid retrieved, and eliminated the need for balloon dilation, thereby reducing the overall procedure time (ranging from 45 to 185 mL) In previous percutaneous peritoneal lavage studies, although 800 mL of saline was instilled, the reported recovered volumes ranged from approximately 5 to 400 mL, with a median of around 60 mL, while achieving sensitivity and specificity comparable to those of staging laparoscopy. In the present study, the recovered lavage volumes were within a similar range to those reported in the literature. Therefore, despite the relatively small fraction of fluid retrieved, it is reasonable to speculate that comparable cytological adequacy may be achievable in human applications of EUS-guided peritoneal lavage.

The EndoSheather is a device-delivery system designed to enable therapeutic devices to be inserted safely and smoothly under endoscopic guidance. It consists of an outer sheath and an inner sheath (dilator), with the tip of the dilator tapered to facilitate easy passage through strictures over a guidewire. In the present study, an appropriate degree of rigidity was also required to penetrate the gastric wall. Additionally, once the inner sheath was removed, the outer sheath became sufficiently flexible to maintain procedural safety. In case 6, despite the addition of side holes, only a small amount of fluid was obtained. This was likely due to the tip of the tube and the side holes being in contact with the peritoneum. Further technical refinements will be required to ensure more consistent and stable fluid retrieval in future procedures. There were several anatomical differences between humans and the porcine models.

First, the waterfall-shaped stomach of the porcine models made endoscopic manipulation somewhat challenging. Additionally, with the pancreas in them being thinner and smaller compared to humans, it was difficult to visualize using the EUS images. We could visualize the splenic vasculature by directing the endoscope toward the posterior wall of the stomach and subsequently observe the edge of the pancreas.

Second, we performed interventions on the omental bursa, which lies between the stomach and pancreas. There was one incident of a mucosal injury that occurred during saline injection, but it was minor, and the procedure was successfully completed following re-puncture. The reason of this submucosal injury was that the tip of the ENBD tube remained within the submucosal layer and failed to penetrate the abdominal cavity. This led to the injection of saline into the submucosal layer. This incident was likely related to the thickness of the porcine gastric mucosa. Because the human gastric mucosa is generally even thicker, a similar event, although probably uncommon, cannot be completely ruled out.

Third, fluoroscopy was not feasible due to the restrictions associated with the animal facility, which resulted in an uncertainty regarding the position of the tip of the ENBD tube. In clinical practice, fluoroscopy with contrast medium would facilitate visualization of the tip of the ENBD tube.

Despite these technical issues, the procedures in this study were performed safely. All the porcine models used in this study resumed eating without any adverse effect on their appetite or appearance of any digestive symptoms. None of the cases had any complications until the time of necropsy, which was performed one week after the procedure. At the time of necropsy, a similar intervention was performed by tracing the endoscopic clip in the gastric wall, for replicating the steps of the procedure. This replication demonstrated that the position of the tip of the ENBD tube in the abdominal cavity was in the appropriate position, which was visually confirmed. This observation supports the safety and effectiveness of EUS-LT. From a diagnostic standpoint, EUS-LT has several limitations compared with staging laparoscopy (SL): it does not allow for visualization of other organs, and liver metastases or peritoneal dissemination cannot be detected, unlike with the conventional method. As this study did not utilize a tumor model, no malignant cells were present in the collected lavage fluid, and therefore no conclusions regarding diagnostic performance can be drawn. In addition, the limited sample size and the inability to perform postoperative blood examinations, which precluded more detailed physiological assessment, should also be considered as limitations of this study. These subgroup analyses should be interpreted cautiously due to the small sample size and were performed to explore technical trends rather than to establish equivalence. Finally, the follow-up period in this study was limited to one week; therefore, longer-term complications such as gastric leak, peritonitis, or adhesion formation could not be excluded and should be evaluated in future studies with extended follow-up durations.

However, EUS-LT could be easily and safely performed at the endoscopic unit during the same session of EUS-FNA. From a translational perspective, this study serves as a preclinical proof of concept demonstrating the technical feasibility and safety of EUS-LT using an in vivo porcine model. Importantly, the procedure relies exclusively on clinically available EUS equipment and accessories, without the need for novel devices. This feature lowers the barrier to clinical translation and facilitates future first-in-human feasibility studies. We believe that EUS-LT has the potential to improve staging accuracy and decrease the number of patients who are considered ineligible for surgery in the future. It may represent a practical and minimally invasive approach for the staging of pancreatic cancer. In addition, EUS-LT can be performed using the Douglas approach, percutaneous routes, and other alternative approaches [29]. Additionally, EUS-LT possesses the advantage that it can be performed at the same sessions with EUS-FNA. This helps in avoiding and preventing unnecessary surgeries (including exclusion of candidates ineligible for surgery) and enables more rapid initiation of subsequent treatments (chemotherapy, endoscopic biliary drainage, etc.) [30]. In contrast to these diagnostic applications, several EUS-guided therapeutic approaches for pancreatic cancer have been reported, including tumor ethanol ablation combined with celiac plexus neurolysis [31]. Our study focuses on a diagnostic staging approach rather than a therapeutic intervention.

Beyond the technical feasibility demonstrated in this study, the clinical relevance of EUS-LT warrants further consideration. As the therapeutic landscape of pancreatic cancer continues to evolve, particularly with the increasing adoption of neoadjuvant therapy, accurate staging has become even more critical. A significant proportion of patients who appear resectable on cross-sectional imaging are ultimately found to have occult peritoneal metastases at the time of surgical exploration. Thus, a minimally invasive technique capable of detecting microscopic peritoneal disease could meaningfully influence treatment decisions and prevent unnecessary laparotomies. In this context, EUS-LT may serve as an intermediate diagnostic modality between conventional imaging and staging laparoscopy, offering cytological information with significantly lower procedural burden.

Additionally, EUS-LT may provide opportunities for future translational research. Lavage fluid obtained from the peritoneal cavity could be used not only for cytological evaluation but also for molecular analyses, such as next-generation sequencing, proteomic profiling, or assessment of extracellular vesicles [32,33,34]. These advanced assays may help identify high-risk biological signatures, facilitate early detection of metastatic disease, or guide individualized therapeutic strategies. Such applications could broaden the utility of EUS-LT beyond staging alone. Another advantage is the procedural accessibility of EUS-LT. Because it can be performed by endoscopists experienced in EUS-guided interventions, the need for surgical resources is minimized. In many institutions where staging laparoscopy is not routinely performed, EUS-LT could expand access to more precise staging. Integration of EUS-LT into the diagnostic workflow may also streamline patient care by consolidating diagnostic steps into a single endoscopic session.

Future studies should aim to evaluate the diagnostic yield of EUS-LT in human subjects, compare outcomes with staging laparoscopy, and determine the optimal lavage volume and technique. Establishing standardized criteria for cytological and molecular analysis will also be essential for broader clinical adoption.

## 5. Conclusions

This preclinical in vivo porcine study demonstrated the technical feasibility and short-term safety of a novel endoscopic ultrasound-guided lavage technique (EUS-LT). While diagnostic performance was not assessed, these findings provide a proof-of-concept supporting further evaluation of EUS-guided peritoneal lavage in future first-in-human feasibility studies.

## Figures and Tables

**Table 1:** Characteristics and Procedural Outcomes of the Ten Porcine Models.

	Case 1	Case 2	Case 3	Case 4	Case 5	Case 6	Case 7	Case 8	Case 9	Case 10
Body weight (kg)	43	45	42	40.4	40.5	37.1	39.8	39.7	36.4	38.4
Procedure success	yes	yes	yes	yes	yes	yes	yes	yes	yes	yes
Intraoperative complications	None	Yes *	None	None	None	None	None	None	None	None
Postoperative Complications	None	None	None	None	None	None	None	None	None	None
Procedure time (minutes)	48	55	25	25	40	45	16	15	23	19
Infusion time (minutes)	25	33	15	5	8	10	5	4	8	7
Injection volume (mL)	800	800	800	800	800	800	800	800	800	800
Amount of recovery (mL)	8	10	170	64	13.5	8	185	110	50	45

* Mucosal injury.

**Table 2:** Comparison of Procedural Performance Across Three Sequential Technique Refinement Groups.

	G1	G2	G3	*p*-Value	Significant Pairwise Differences (Holm-Adjusted)
Body weight (kg)	44.0 (43.5–44.5)	40.5 (39.0–41.0)	39.1 (37.5–39.6)	0.094	none
Procedure time (min)	51.5 (49.8–53.3)	32.5 (25.0–42.5)	17.5 (15.5–21.0)	0.018	G1 > G2, G1 > G3
Infusion time (min)	29.0 (27.0–31.0)	9.0 (6.5–11.3)	6.5 (5.0–7.5)	0.022	G1 > G2, G1 > G3
Amount of recovery (mL)	9.0 (8.5–9.5)	13.8 (10.8–92.3)	77.5 (47.5–122.5)	0.031	G1 < G2, G1 < G3 (G2 vs. G3: n.s.)

G1; Group1 (cases 1–2) Median (IQR), G2; Group2 (cases 3–6) Median (IQR), G3; Group3 (cases 7–10) Median (IQR).

## Data Availability

The data supporting the findings of this study are available from the corresponding author upon reasonable request.
